# Transpedicle body augmenter for vertebral augmentation in symptomatic multiple osteoporotic compression fractures

**DOI:** 10.4103/0019-5413.62016

**Published:** 2010

**Authors:** Allen Li, Kung-Chia Li, Ching-Hsiang Hsieh

**Affiliations:** Department of Biology, Johns Hopkins University, Baltimore, MD 21218, USA; 1 Department of Orthopedic Surgery, Chiayi Yang-Ming Hospital, Republic of China; 2 Institute and Faculty of Physical Therapy, National Yang-Ming University, Taipei, Taiwan, Republic of China

**Keywords:** Vertebral compression fracture, osteoporosis, transpedicle body augmenter

## Abstract

**Background:**

Multiple osteoporotic vertebral compression fractures (VCFs) have been treated with polymethylmethacrylate augmentation; however, there are cement complications and long-term fracture healing that are unknown. Transpedicle body augmenter (a porous titanium spacer) has been reported as an internal support to reconstruct the vertebral body combining short-segment fixation in burst fracture and Kümmell's disease with cord compression. Transpedicle body augmenter for vertebral augmentation (TpBA) also has been reported successfully in treating single painful VCF and vertebral metastasis lesions including pending fractures and pathologic compression fractures. To test the hypothesis that TpBA can effectively and safely treat the symptomatic multiple VCFs, this retrospective study was done by analyzing the radiographic and clinical results.

**Materials and Methods:**

We retrospectively reviewed clinical and radiographic results of TpBA for symptomatic multiple (more than two levels) VCFs in 62 patients with a total of 236 levels, i.e. 3.8 VCFs per patient. Manual reduction and TpBA via paramedian incisions with blunt dissection were done. One incision was made for two continuous levels and alternative side was selected for next incision. Mean age was 74.3 years (range, 62-87 years), and female-male ratio was 5.2:1. Anterior vertebral height and wedge angle by radiographic findings were measured at preoperative, initial follow-up and final follow-up. Clinical results were assessed by questionnaires and clinical observations. By July 2008, 58 patients returned to answer the questionnaire including quantification of pain on the visual analog scale, the response to operations (better, same, or worse after operation), returned to their pre-fracture function (yes/no) and satisfaction (a scale of 0 = completely dissatisfied to 10 = completely satisfied).

**Results:**

The mean symptom duration was 7 months, and follow-up, 48 months. The average operation time was 21 min per level, blood loss was 74 cc per level and hospitalization was 4.4 days. No patient had neurological deterioration. There was no dislodgement of implant in the final visit. Forty-eight patients (77.4%) could walk within 6-8 h after operation and the others, within 24 h. The anterior vertebral restoration was 7.3 mm initially and 6.2 mm at final follow-up. Wedge angle correction was 10.4° initially and was 9.3° at final follow-up. Pain, by the visual analog scale, was 8.5 preoperatively, 2.7 at day 7 follow-up and 2.9 at final follow-up. By the questionnaire, 52 of 58 respondents reported a decrease in discomfort after TpBA and 48 of 58 patients reported a return to normal activity after operation. The final satisfaction rate was 89.7%.

**Discussion:**

The symptoms of multiple osteoporotic compression fracture may be due to unstable fracture, radiculopathy, and global traumatic kyphosis with posture changes, which can be corrected by multiple TpBA. The transpedicle body augmenter was initially stabilized by the sinking and locking mechanism and finally by bone ingrowth.

**Conclusions:**

TpBA via a minimally invasive method led to early and medium-term clinical improvements and anatomic restoration of multiple symptomatic VCFs.

## INTRODUCTION

Vertebral compression fractures (VCFs) cause many significant problems, including pain,^1^ spinal deformity,^2^ reduced pulmonary function,^3^ reduced mobility,^4^–^6^ and an increase in mortality.^7^ Traditional treatments for patients with VCFs include bed rest, analgesia, and bracing, which are unable to restore spinal alignment and, furthermore, reduced mobility can increase the rate of demineralization.^2^ All these symptoms can be exaggerated in multiple VCFs.

Although polymethylmethacrylate (PMMA) vertebroplasty or kyphoplasty has been reported to treat VCFs with good clinical results^8^–^10^ and correction of deformity,^11^–^13^ some complications have been reported,^14^–^20^ including cement extravasation,^16^,^21^–^23^ pulmonary embolism,^24^,^25^ deep infection,^26^ postprocedure acute respiratory distress syndrome,^27^ palsy,^28^ and death.^21^,^29^ To minimize these complications, modified techniques have been carried out, with a modest degree of sucess.^30^–^32^

Another minimally invasive method, i.e. transpedicle body augmentation (TpBA) with titanium spacers was reported to treat VCFs effectively.^33^ Manual reduction and TpBA were initially applied to restore burst fractures and Kümmell's disease with cord compression.^34^–^37^ The concept is that a fractured body can be reduced by manual reduction and reconstructed with TpBA via a posterior approach. The collapsed body can be supported internally to allow long- term fracture healing. In treating single VCF with TpBA, the average operation time was 26.1 min, blood loss 92 cc and hospitalization 2.3 days. All the patients started walking within 24 h with a final satisfaction rate of 93.4%.^33^ The TpBA had no implant dislodgement due to the sinking and locking mechanism after patient ambulance. The success experience is also reported in pathologic vertebral fracture.^38^ However, to date, there was no report of the usage of TpBA to treat symptomatic multiple VCFs, which involved more cardiopulmonary and gastroenteric problems secondary to global spinal deformity. With the hypothesis that TpBA can effectively and safely treat the symptomatic multiple VCFs, a retrospective study was done to evaluate the effects of TpBA for symptomatic multiple (≥3 levels) VCFs in 62 patients with 236 levels.

## MATERIALS AND METHODS

A retrospective study was performed on 68 consecutive patients affected by symptomatic multiple (≥3 levels) ver tebral compression fracture (VCFs) between July 2003 and June 2005. The inclusion criteria for this report required: neurologic functional status limited to Frankel E,^39^ multiple levels, nonpathological fractures and unresponsiveness to nonoperative methods. The mean duration of symptoms was 7 months (range, 17 days to 40 months). Symptomatic levels were identified by correlating the clinical findings with marrow signal changes consistent with compression fractures in magnetic resonance images. Perioperative variables and TpBA complications were recorded and analyzed. The follow-up rate was 91%. Five patients died of unrelated medical illnesses and one patient was lost to follow-up. These six patients were excluded from this retrospective study. Finally, in this study, there were 236 levels [Table 1] in these 62 patients, i.e. 3.8 VCFs per patient. The mean patient age was 74.3 years (range, 62-87 years), and the women to men ratio was 5.2 : 1. The mean follow-up period was 48 months (range, 36-62 months).

**Table 1:** Distribution of compression fractures in this series

Level	Number
T7	8
T8	9
T9	10
T10	21
T11	31
T12	52
L1	53
L2	28
L3	14
L4	5
L5	5
Total	236

A standard monitor system was set up for all patients. All the patients received general anesthesia in this series. General anesthesia was induced by administration of fentanyl (1.5-2 mcg/kg), propofol (1-2 mg/kg) and succinylcholine (1-2 mg/kg). Nimbex provided muscle relaxation. Anesthesia was maintained with inhalation drug (desflurane) and titrated to maintain hemodynamic stability.

All patients initially underwent manual reduction.^33^–^38^ Acute and nonunion compression fractures were reduced easily by the manual procedure, leaving a substantial bony defect in the vertebral body. C-arm fluoroscopy was used to locate the fracture site and monitor the insertion of transpedicle body augmenter (a porous titanium spacer, Merries International Inc., Taipei, Taiwan).

**Figure 1:**
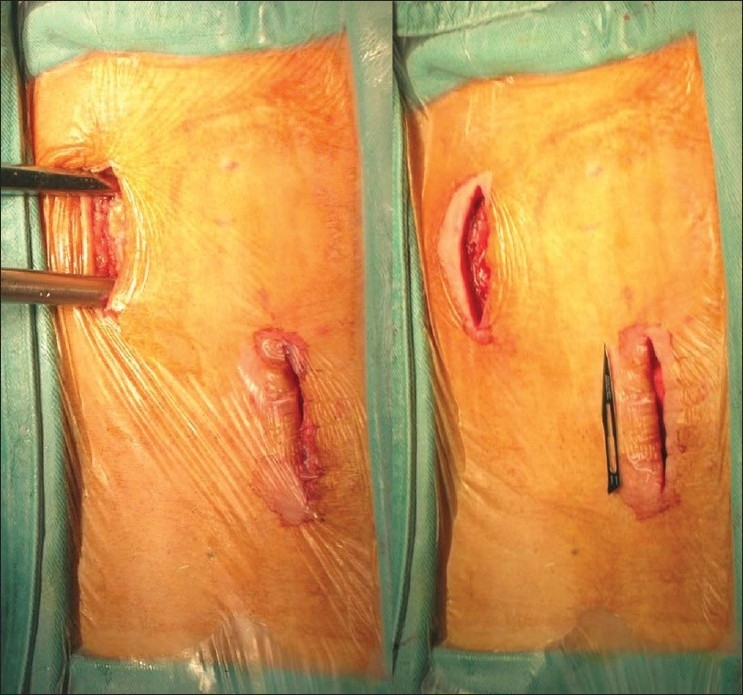
Clinical photograph showing one paramedian incision made for T11-T12 compression fractures and an alternative side was selected for next incision for L1-L2 lesions

The TpBA was performed through paramedian incisions of about 3 cm in length with muscle splitting and blunt dissection. One incision was made for two continuous levels and alternative side was selected for next incision [Figure 1]. A guiding pin was first inserted and confirmed by C-arm fluoroscopy. Unilateral pedicle tunnel into the vertebral body was made by an awl, followed by serial custom-made dilators to prepare for the passage of the transpedicle body augmenter. The concept of safe zone^34^ for TpBA is that lateral and superior cortices of the pedicle can be violated without neural or vascular injury. The softness of osteoporotic pedicle allows for the lateral and superior dilation of pedicles without any brittle breakage. The bony defect in the fractured vertebral body was filled through the pedicle tunnel^40^ with mercerized allograft. Then a transpedicle body augmenter was inserted into the vertebral body through the pedicle tunnel. No Hemovac drainage was needed. Patients wore a thoracolumbar brace for 3 months. After discharge, patients were followed up regularly. Operation time, blood loss, hospitalization, and complications were documented.

Preoperative spine radiographs were taken with the patient in the supine position. Serial radiographs (supine anteroposterior and lateral radiographs centered on the involved region) were taken postoperatively. Flexion and extension radiographs were taken after 1 year and at the final follow-up visit to identify the integration of transpedicle body augmenter and vertebral bone. In the radiographic analysis, wedge angle of the fractured vertebral body was measured as described previously by Verlaan *et al*.^41^ The radiographic parameters were measured on neutral thoracolumbar radiographs of 62 patients before the operation, immediately postoperatively and at the final follow-up. All digitization and measurements were done by the same experienced research assistant in EBM viewer software (EBM Technologies Inc., Taipei, Taiwan) with an accuracy of ±0.1 mm. Repeat measurements of the same vertebral levels after a 10-day interval with the same observer demonstrated an error of ± 1.2 mm in height and ± 2.2° in wedge angle.

Clinical results were assessed by questionnaires and clinical observations. Between May 2008 and July 2008, each patient was called or mailed to return to our institution in person and answer the questionnaire. Finally, 58 patients (93.5%) completed the questionnaires. Other patients were not able to answer the questionnaire due to memory recall issues. The patients were asked to quantify their degree of pain on the visual analog scale (VAS) (0 = no pain; 10 = worst pain) before operation, 3, 7, and 30 days after operation, and at present. All these 58 patients independently completed a questionnaire to describe if they felt better, the same, or worse after operation; whether they had returned to their pre-fracture function (a yes/no question); how satisfied they felt with this operation (a scale of 0 = completely dissatisfied to 10 = completely satisfied).

One-way ANOVA was used for statistical analysis of pain VAS among the data of pre-operative, 3-day, 7-day, 30-day, and final follow-up. All the data presented were reported as mean±standard deviation. The level of statistical significance was set as *P*<0.05

## RESULTS

All 62 patients tolerated the multiple TpBAs well with limited blood loss and operation time. All patients subjectively reported immediate relief of their typical fracture pain, and no patients complained of worse pain at the treated levels. Hospitalization was 4.4±1.8 days (range 2-7 days). The operation time was 21.3±6.2 min (range 14-35 min) per level. The blood loss was 74±55 cc (range, 40-200 cc) per level. All patients could walk 3-24 h after surgery. Forty-eight patients (77.4%) could walk within 6–8 h after operation and the other within 24 h. Postoperative complications included one pneumonia and two skin edge necroses, which received debridement. No deep wound infection was found. Neural deterioration and root irritation were not found after TpBA. No dislodgement or loosening of any transpedicle body augmenter was found at the final visit. There were 11 new (8 adjacent and 3 remote) compression fractures (17.7%) during follow-up. Seven patients underwent TpBA. The other four patients refused further operations.

**Figure 2:**
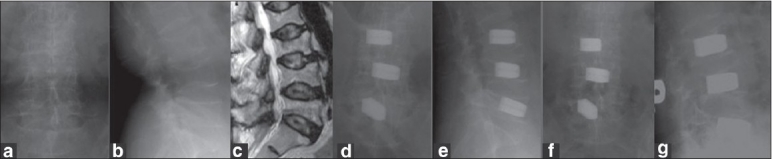
(a-c) Radiograph (anteroposterior and lateral) and T2W sagittal MRI in a 62-years-old female who suffered severe low back pain and was unable to upright her back without root signs showed L3–L5 osteoporotic compression fractures without root compression. (d,e) Radiograph (anteroposterior and lateral view) revealed three-level TpBA vertebroplasties without laminectomy immediately after operation. (f,g) Radiograph (anteroposterior and lateral views) at 4-year follow-up demonstrated good status of TpBA vertebroplasties

**Figure 3:**
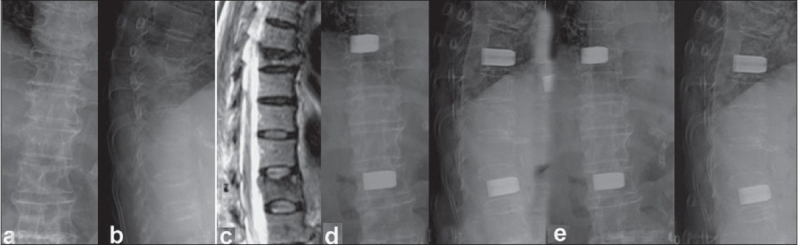
(a-c) Radiograph (anteroposterior and lateral) and T2W sagittal MRI in an 80-years-old female suffered severe mid back pain with T8 costal neuralgia radiating to anterior chest wall demonstrated T8 and T12 osteoporotic compression fractures without cord compression. (d) Radiograph (anteroposterior and lateral view) showed two-level TpBA vertebroplasties immediately post-operatively. The back pain and costal neuralgia subsided postoperatively (e) Radiograph (anteroposterior and lateral views) at 38-month follow-up illustrated well restoration and maintenance of the vertebrae

**Figure 4:**
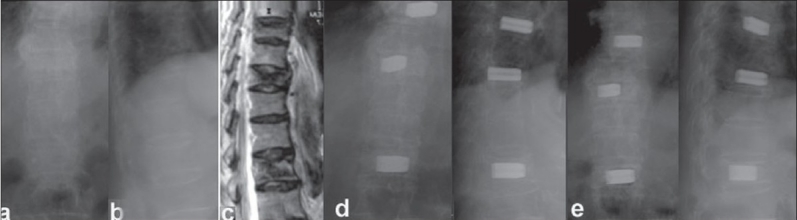
(a-c) Radiograph (anteroposterior and lateral) and T2W sagittal MRI in an 84-years-old female suffered intolerable mid back pain with T8 and T10 costal neuralgia radiating to anterior chest wall showed T8, T10 and L1 osteoporotic compression fractures without cord compression. (d) Radiograph (anteroposterior and lateral view) revealed 3-level TpBA vertebroplasties at initial follow-up. The back pain and costal neuralgia relieved postoperatively. (e) Radiograph (anteroposterior and lateral views) at 30- month follow-up confirmed the good results of TpBA vertebroplasty

The restoration of anterior vertebral height and wedge angle correction was achieved and maintained well by TpBA [Figures 2–4]. The anterior vertebral restoration was 7.3±3.2 mm initially and 6.2±2.7 mm at final follow-up. Wedge angle correction was 10.4°±2.2° initially and was 9.3°±2.0 at final follow-up. There was only 1.1 mm loss of anterior body height correction and 1.1° loss in wedge angle correction in the final follow-up, which was within the measurement deviation. However, due to the mixed distributions at thoracic kyphotic and lumbar lordotic regions in this series, the global sagittal deformity is a concept of multiple wedge fractures rather than a real data set.

The early and medium-term clinical outcomes were satisfactory. Three patients had unstable gait at the final visit due to two cerebral strokes and one Parkinsonism, which we considered unrelated to the TpBA. Other patients remained ambulatory. The sequential average pain VAS in 58 respondents at pre-operative, 3-day, 7-day, 30-day and final follow-up were 8.5±1.2, 3.6±1.3, 2.7±1.2, 2.3±0.9 and 2.9±2.2, respectively, which revealed a reduction in pain intensity after TpBA (One-way ANOVA, *P*<0.001). By the questionnaire, 52 of 58 respondents reported a decrease in discomfort after TpBA, and 48 of 58 patients reported a return to normal activity after operation. Thirty- five patients (60.3%) were satisfied (5-8 on a scale of 0-10) and 17 patients (29.4%) were very satisfied (9-10, on a scale of 0-10) after TpBA. Only 6 (10.3%) of the 58 respondents were dissatisfied due to cerebral strokes (2 patient), Parkinsonism (1 patient) and new compression fractures without further treatment (3 patients). The average satisfaction score was 7.2±2.2.

## DISCUSSION

The TpBA provides a new option to treat symptomatic multiple VCFs. Although cement ver tebroplasty 22,24 or kyphoplasty have achieved good results in treating VCFs,^11^–^13^ severe complications have been reported 18,19 and long-term results are currently unknown. Reconstruction with structural bone graft and instrumentation may be performed in patients with concurrent spinal instability or neurological compromise from an anterior or posterior approach; however, the success of these techniques is limited by the patient's poor bone quality and general medical condition. Manual reduction and TpBA have been reported to restore and maintain the vertebra in VCFs and pathologic vertebral compression fractures successfully.^33^,^38^ The concept is that a fractured body can be reduced by manual reduction and reconstructed with a transpedicle body augmenter via a posterior paramedian approach. The collapsed body can be supported internally to allow long-term fracture healing. TpBA had no implant dislodgement due to the sinking and locking mechanism after patient ambulance. Because it is minimally invasive, TpBA is ideal for geriatric patients. Furthermore, all cement-related complications in vertebroplasty or kyphoplasty can be prevented by this procedure. Our retrospective study showed that multiple TpBA were well-tolerated and effective procedures for the treatment of symptomatic multiple VCFs.

In our study, the use of TpBA as a mean to treat symptomatic multiple VCFs achieved good medium-term clinical results. TpBA attains excellent pain relief and restored vertebral height. It may be related with restoration of vertebral body, decrease of false motion of fractured segment, decompression of ner ve roots and fracture healing. Compared with a single VCF, a patient with multiple VCFs had more gastroenteric and cardiopulmonary complaints, and secondary posture changes due to global traumatic kyphosis, which decreased after TpBA.

TpBA may provide another option to treat symptomatic multiple VCFs. Although PMMA vertebroplasty is successful for pain relief in VCFs,^17^,^23^,^42^,^43^ this technique does not attempt to restore the height of the collapsed vertebral body. The major problem of PMMA vertebroplasty is cement extravasation, which can be as high as 72.5% in metastases^16^ and 65% in osteoporotic fractures.[Bibr CIT23] Kyphoplasty also has a leakage rate of 10.2% in the osteoporotic group and 8.3% in the tumor group.^44^ The cement leakage may cause neural compression and induce subsequent adjacent fracture if leakage occurs in disc spaces.^45^ TpBA with bone graft theoretically allows fracture healing in the long term. The high ratio of the patients with subsequent fractures willing to receive second TpBA suggests the effect of this procedure and its acceptance by the geriatric patients.

The medium-term subsequent fracture rate after our multiple TpBA was 17.7%, which is similar to the reported natural course for an osteoporotic spine. New compression fractures have been reported as 12% in the subsequent year in patients with one previous compression fracture and 24% in those with two previous fractures.^46^ There is a wide range (3–29%) of reported subsequent fractures after kyphoplasty.^45^,^47^ Harrop *et al*, reported that the incidence of subsequent fractures, mean 11 months after kyphoplasty, was 15.1% per procedure (34 of 225 procedures) or 22.6% per patient (26 of 115 patients).^48^ Compared with these results, multiple TpBAs did not increase the rate of new compression fractures.

Multiple TpBAs can reduce the global deformity by corrections of multiple procedures. Multiple compression fractures induce more pain^49^ and larger traumatic kyphosis by accumulation of individual wedge shape of involved vertebrae and secondary adjacent disc degenerative changes, which may impact pulmonary-cardiac and gastroenteric functions. The advantage of TpBA is that it is minimally invasive with limited blood loss. In addition, less operation time is required. Thus TpBA can be repeated for multiple injured vertebrae, allowing for the correction of global deformity within one-stage operation. In addition, because these procedures do not have instrumentation or interbody fusion, TpBA therefore is suitable for geriatric and vulnerable patients.

Although TpBA was performed for multiple vertebrae, no dislodgment of implant was noted. This is due to the mechanism of sinking and locking of transpedicle body augmenter within the vertebra.^33^,^38^ Cancellous bone within the vertebrae allows the loaded transpedicle body augmenter to sink while the patient is in an upright posture. The transpedicle body augmenter will be locked by the peripheral residual cortices and would be unlikely to move anywhere. After bone ingrowth, the locked transpedicle body augmenter will be further stabilized. In the early stage, transpedicle body augmenter is an internal supporter to maintain body height and ensure cancellous bone regeneration. Finally, transpedicle body augmenter also works in an emergency to prevent new crushing of the vertebral body when it is subjected to an external force.

TpBA was found to associate with an improvement in pain, restoration of anterior body height, correction of wedge angles of each vertebra and overall sagittal alignment of whole spine for patients with multiple vertebral compression fractures. TpBA can be applied in geriatric patients, even in patients with poor bone quality or vulnerable medical conditions. However, some limitations of our study should be mentioned. Because the study was retrospective, accuracy of VAS measures may be compromised due to recall bias. Evaluation was not blinded in this study, which would impart observer bias to the final report; however, because the transpedicle body augmenter was clearly visible in radiograms, blinded evaluation of radiographic parameters would be impossible. Further, the clinical results were evaluated by the authors who were not blinded. Another limitation to our study is its single-hospital report. Multicentric trials enrolling larger patient populations may provide further information.

Because TpBA provides body reconstruction without instrumentation or cement usage along with short operation time, limited blood loss, and minimally invasive nature, TpBA is a safe and effective operation to restore and stabilize symptomatic multiple osteoporotic compression fractures.
